# Association between Abnormal Antenatal Doppler Characteristics and Gastrointestinal Outcomes in Preterm Infants

**DOI:** 10.3390/nu14235121

**Published:** 2022-12-02

**Authors:** Silvia Martini, Mariarosaria Annunziata, Anna Nunzia Della Gatta, Arianna Aceti, Marica Brunetti, Gianluigi Pilu, Giuliana Simonazzi, Luigi Corvaglia

**Affiliations:** 1 Neonatal Intensive Care Unit, IRCCS AOU S. Orsola, 40138 Bologna, Italy; 2 Department of Medical and Surgical Sciences, University of Bologna, 40138 Bologna, Italy; 3 Obstetrics Unit, Department of Obstetrics and Gynecology, IRCCS AOU S. Orsola, 40138 Bologna, Italy; 4 Department of Pediatric Sciences, Catholic University Medical School, 00168 Rome, Italy

**Keywords:** preterm infants, antenatal Doppler, umbilical artery, middle cerebral artery, ductus venosus, necrotizing enterocolitis, feeding intolerance, gastrointestinal outcome, nutrition

## Abstract

Antenatal Doppler disturbances are associated with fetal hypoxia and may induce a brain-sparing vascular redistribution at the expense of splanchnic circulation, possibly predisposing to gut complications. We aimed to compare several gastrointestinal outcomes among very-low-birthweight (VLBW) preterm infants with different antenatal Doppler features. VLBW infants born between 2010–2022 were retrospectively included and stratified into the following clusters based on antenatal Doppler characteristics: normal Doppler (controls); absent or reversed end-diastolic flow in the umbilical artery (UA-AREDF) alone or also in the ductus venosus (UA+DV-AREDF); and abnormal Doppler with or without brain-sparing redistribution. The following outcomes were evaluated: time to reach full enteral feeds (FEF), feeding intolerance (FI), necrotizing enterocolitis (NEC), and spontaneous intestinal perforation (SIP). Overall, 570 infants were included. Infants born following UA+DV-AREDF had significantly higher FI, NEC, and SIP rates and achieved FEF later compared to controls. Increased FI prevalence and a longer time to FEF compared to controls were also observed among UA-AREDF infants and in the presence of brain-sparing redistribution, which also increased NEC rates. Antenatal Doppler abnormalities exacerbate the gastrointestinal risks of preterm infants. Detailed knowledge of Doppler features can aid in identifying those at highest risk of intestinal complications who may benefit from tailored enteral feeding management.

## 1. Introduction

Placental vascular dysfunction results from an abnormal trophoblast invasion of spiral arteries, which leads to an increased resistance of the placental vessels and a reduced uteroplacental blood flow [1]. This condition is often associated with intrauterine growth restriction (IUGR) in fetuses [1] and represents an independent risk factor for increased morbidity and mortality among preterm neonates [2,3,4].

Doppler ultrasonography of the umbilical artery (UA), middle cerebral artery (MCA), and ductus venosus (DV) is the gold-standard procedure to assess the extent of placental vascular insufficiency and the resulting impairment of fetal circulation [5,6,7,8].

The early Doppler sign of a pathological increase of placental vascular resistance is a decrease in the blood flow in the UA, which can progress to absent or reversed end-diastolic flow (UA-AREDF) [2,9]. This, in turn, contributes to increasing the fetal ventricular afterload, which affects fetal perfusion and leads to chronic hypoxia and lactic acidosis. In order to maintain adequate perfusion of such vital organs as the brain, the heart, and the adrenal glands, fetuses with an impaired antenatal Doppler may develop a “brain sparing” response, characterized by decreased vascular resistance in the cerebral arteries and by the diversion of blood flow from the splanchnic circulation towards these vessels [10]. A reduced pulsatility index (PI) in the MCA indicates the presence of this vascular remodeling. However, if these compensatory changes are inadequate to sustain fetal hemodynamics or do not occur, right atrial pressure may progressively increase, reducing the venous return and affecting blood flow velocity in the DV. The Doppler abnormalities that characterize this failing phase range from an increased PI to an absent or inverse α-wave in the DV (DV-AREDF) and indicate a progressive compromise of fetal hemodynamics [10,11,12,13,14].

Fetal splanchnic hypoperfusion first results from the chronic hypoxia associated with placental insufficiency and is further worsened by the compensatory hemodynamic changes associated with the brain-sparing phenomenon. This may contribute to increasing the risk of ischemic gut complications in such a vulnerable population as preterm neonates, who are intrinsically prone to developing adverse gastrointestinal (GI) outcomes due to their functional and anatomic gut immaturity [15]. Current literature has reported increased rates of adverse GI outcomes in IUGR infants [4,16,17,18,19,20], but evidence on the contribution of specific antenatal Doppler abnormalities in determining this increased risk is scanty; in particular, only a couple of studies have assessed the possible correlation between NEC development and the features and severity of antenatal Doppler abnormalities [20,21].

This study aims to fill these gaps by comparing GI and nutritional outcomes in very-low-birth-weight (VLBW) preterm infants with different features of antenatal Doppler impairment and evaluating the impact of the severity of Doppler abnormalities and the vascular remodeling associated with brain sparing.

## 2. Materials and Methods

This retrospective observational study included infants admitted to the Neonatal Intensive Care Unit (NICU) of IRCCS AOU Bologna (Italy), between January 2010 and March 2022, according to the following criteria: gestational age <32 weeks and/or a birth weight <1500 g, availability of antenatal Doppler data. Infants with major congenital abnormalities deceased within the first week of life or transferred to other hospitals before achieving any of the study outcomes were excluded.

This study was conducted in conformity with the principles and regulations of the Helsinki Declaration. The protocol was approved by the Ethics Committee “Area Vasta Emilia Centro—AVEC”, Bologna, Italy (protocol no. 221/2022/Oss/AOUBo).

Based on the available data on the features of antenatal Doppler, the study population was stratified into two grouping systems according to the following classifications based on the vessels involved by the Doppler disturbances or on the presence/absence of brain-sparing: (1) normal antenatal Doppler, abnormal UA Doppler (UA-AREDF), abnormal DV Doppler (UA+DV-AREDF); (2) normal antenatal Doppler, impaired antenatal Doppler with brain-sparing, impaired antenatal Doppler without brain-sparing [2].

The following outcomes were compared between the study groups: time to reach full enteral feeds (FEF, defined as an adequate enteral feeding tolerance of 150 mL/kg/day) [22]; duration of parenteral nutrition (PN); prevalence of feeding intolerance (i.e., defined as withholding of enteral feeding ≥24 h because of two or more of the following suggestive signs: abdominal distension, absent bowel sounds, persistent gastric residuals [GR], GR volume >2 mL/kg of body weight or greater than half the volume of the previous feed, bilious or bloody GR and/or bloody stools [16,23]); necrotizing enterocolitis (any stage and Bell’s stage ≥2a) [15]; spontaneous intestinal perforation (SIP) [24]; mortality rates after the first week of life; length of hospital stay.

Since, during the study period, a human milk bank was established in our local hospital, the feeding type administered to the study infants during their hospital stay (exclusive human feeding vs. any formula feeding) was noted and included in the multivariable analysis. Apgar scores at 5′ and the need for invasive respiratory support during the hospital stay were also collected.

### Statistical Analysis

Based on the prevalence of NEC (any stage) observed at IRCCS AOU Bologna over the last 5 years, a minimum sample size of 241 was estimated using G*Power software v. 3.1.9.6, with power = 0.80, α = 0.05 and effect size w = 0.2.

Data distribution was analyzed using a Shapiro–Wilk test. Numerical variables were summarized as mean ± standard deviation or median (interquartile range), as appropriate; categorical variables were summarized as frequencies and percentages. Since the data did not follow a normal distribution, non-parametric methods were used for statistical analysis. The comparison of the study outcomes between the different Doppler groups (UA-AREDF vs. UA+DV-AREDF vs. normal Doppler; brain sparing vs. no brain sparing vs. normal Doppler) was performed using a chi-square or Fisher’s exact test for categorical variables and a Kruskal–Wallis test with Bonferroni post-hoc correction for continuous variables.

The study outcomes that differed significantly among the Doppler groups at the univariate analysis were used to build different generalized linear models (GLMs) in which the type of Doppler alteration was included as a factor and potential confounders, including the neonatal variables that differed among groups, as covariates. The variance inflation factor (VIF) was used to assess multicollinearity between the model terms; a VIF < 5 indicates a low correlation of that predictor with other predictors, a value between 5 and 10 indicates a moderate correlation, while VIF values > 10 are a sign for high, non-tolerable correlation of model predictors. A *p* value < 0.05 was considered statistically significant.

Statistical analysis was performed using Statistical Package for Social Science (SPSS) software, version 27 (IBM Corp. Released 2019. IBM SPSS Statistics for Windows, IBM Corp: Armonk, NY, USA). The significance level was set at *p* < 0.05.

## 3. Results

**Figure 1:**
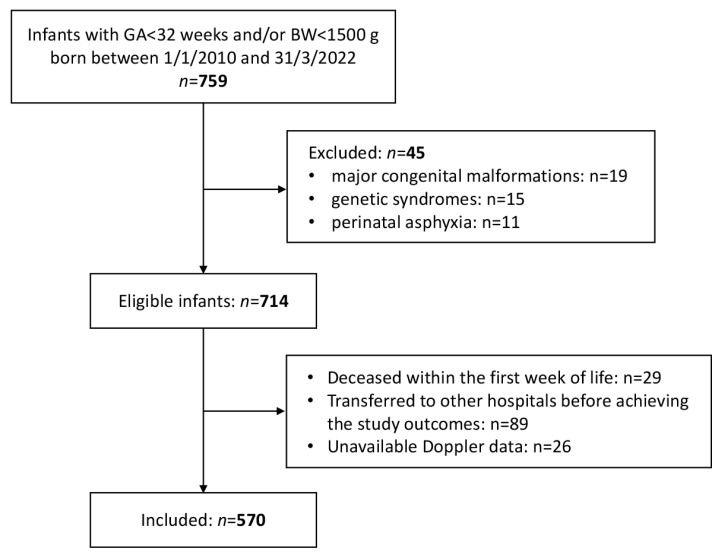
Flow-chart of the study enrolment.

As shown in the enrolment flow-chart (Figure 1), 570 infants were included in the study.

Antenatal Doppler abnormalities were observed in 124 out of 570 infants (21.8%). Of these, 97 (78.2%) had evidence of UA-AREDF and 27 (21.8%) of UA+DV-AREDF. Brain sparing remodeling occurred in 54 (43.5%) of the 124 neonates with evidence of abnormal antenatal Doppler and, in particular, in 21 out of 27 (77.8%) infants with UA+DV-AREDF and in 33 out of 97 (34.0%) infants with UA-AREDF. Infants with normal antenatal Doppler (*n* = 446, 78.2% of the whole study cohort) served as controls.

### 3.1. UA-AREDF vs. UA+DV-AREDF vs. Controls

Clinical characteristics of infants born following UA-AREDF or UA+DV-AREDF and controls are detailed in Table 1. Infants with UA-AREDF had significantly higher GA compared to both UA+DV-AREDF (*p* = 0.007 at post-hoc comparison) and controls (post-hoc *p* = 0.004), while no difference was observed between the latter groups. Compared to the control group, BW and the related z-score were significantly lower in infants with UA-AREDF and UA+DV-AREDF (*p* < 0.001 for all post-hoc comparisons). In the latter group, BW was also significantly lower than in the UA-AREDF group (post-hoc *p* = 0.045). The prevalence of a BW < 10th centile, which defines small gestational age (SGA) neonates, differed significantly between the study groups, being 53.6% in the UA-AREDF group, 51.9% in the UA+DV-AREDF group, and 13% among controls.

The prevalence of maternal hypertension was significantly higher in the UA-AREDF and UA+DV-AREDF groups compared to controls (47.4% and 33.3%, respectively, vs. 20.6% of the control group). The percentage of infants in the UA-AREDF group who received a complete course of antenatal steroids significantly differed between the study groups (*p* = 0.002), being highest in the UA-AREDF (86.5%) and lowest in the UA+DV-AREDF group (63%). Notably, all the infants with evidence of antenatal Doppler alterations were born by cesarean section.

Table 2 illustrates the study outcomes among the 3 groups and the results of between-group comparisons. The prevalence of FI was significantly higher in the UA-AREDF and UA+DV-AREDF groups compared to controls (41.2%, 66.7%, and 28.3%, respectively). Overall, NEC incidence significantly differed among the study groups, being highest in UA+DV-AREDF infants; however, when only NEC cases ≥ 2a Bell’s stage were considered, the between-group difference did not reach statistical significance. SIP was significantly more frequent in the UA+DV-AREDF group compared to UA-AREDF and control infants (14.8% vs. 0% and 1.3%, respectively). Infants with UA+DV-AREDF also had significantly higher mortality rates than those with UA-AREDF and controls (14.8% vs. 1% and 0.2%, respectively). Compared to controls, UA+DV-AREDF infants took a longer time to achieve FEF (*p* = 0.037 at post-hoc comparison), requiring PN for longer periods (post-hoc *p* = 0.010), while no significant difference was observed either between UA+DV-AREDF and UA-AREDF or between the latter infants and controls. Furthermore, infants with UA+DV-AREDF had a longer hospitalization than UA-AREDF infants (post-hoc *p* = 0.049) and controls (post-hoc *p* = 0.003).

In order to adjust the observed results for possible confounders and assess the independent effect of the UA and DV Doppler alterations on FI, NEC development, and time to FEF, specific multivariable GLM models were built (see Table 3). Since no SIP cases were observed in the UA-AREDF group, this outcome was not included in this analysis. Moreover, due to the significant collinearity observed between UA/DV-AREDF and the presence/absence of brain sparing (VIF > 6 for both covariates), only UA and DV doppler alterations were included in these GLMs; no collinearity issues were observed among the other variables included in these models (VIF < 2).

Time to FEF was significantly higher in the presence of UA+DV-AREDF (β = 15.529, 95% CI 8.991–22.066; *p* < 0.001) and UA-AREDF (β = 4.385, 95% CI 0.848–7.922; *p* = 0.015) compared to controls. An association between increasing time to FEF, decreasing GA (β = 2.894, 95% CI 2.279–3.509; *p* < 0.001), and the need for invasive respiratory support (β = 11.286, 95% CI 7.635–14.936, *p* < 0.001) were also observed, while no significant effects were observed for complete antenatal steroids administration, formula feeds during hospital stay, and year of birth.

FI significantly increased in the presence of antenatal UA+DV-AREDF (OR = 5.622, 95% CI 2.230–14.170; *p* < 0.001) and, to a lesser extent, UA-AREDF (OR = 2.281, 95% CI 1.387–3.749; *p* = 0.001) compared to controls. A lower GA (OR = 1.262, 95% CI 1.149–1.385; *p* < 0.001) and formula feeding (OR = 1.953, 95% CI 1.207–3.161; *p* = 0.006) were also independently associated with higher FI rates, while the remaining covariates showed no significant effects.

NEC prevalence (at any stage) was significantly increased by antenatal UA+DV-AREDF (OR = 4.311, 95% CI 1.425–13.044; *p* = 0.010) but not by UA-AREDF. Formula feeding (OR = 2.725, 95% CI 1.090–6.807; *p* = 0.032) and the need for invasive respiratory support (OR = 2.409, 95% CI 1.094–5.306; *p* = 0.029) were also significantly associated with NEC development. No association was observed for the remaining covariates.

### 3.2. Abnormal Antenatal Doppler with Evidence of Brain-Sparing vs. no Evidence of Brain-Sparing vs. Controls

Clinical characteristics of the study groups are provided in Table 4. GA in AREDF infants without brain sparing was slightly but significantly higher than controls (*p* = 0.027 at the post-hoc comparison). Compared to controls, AREDF infants with and without brain sparing had a significantly lower BW and BW z-score (*p* < 0.001 for all post-hoc comparisons). The SGA prevalence was similar in AREDF infants with and without brain sparing (50% and 55.7%, respectively), but significantly higher than in the control group (13%). Similar findings were observed for the prevalence of maternal hypertension, which was significantly higher in AREDF infants with and without brain sparing compared to controls (44.4%, 44.3%, and 20.6%, respectively), and for the antenatal administration of a complete steroid course (79.6%, 82.9%, and 71.7%, respectively).

Table 5 reports the results of the univariate comparison of the study outcomes among the three groups. FI was significantly more frequent among AREDF infants with and without brain sparing compared to controls (50%, 44.3%, and 28.3%, respectively). The prevalence of overall NEC cases significantly differed among the study groups, being highest in the presence of brain sparing remodeling (18.5%). The latter group and, to a lesser extent, AREDF infants without brain-sparing, also showed increased mortality rates compared to controls (5.6%, 2.8%, and 0.2%, respectively). Finally, AREDF infants with brain sparing evidence required a longer hospitalization compared to controls (*p* = 0.020 at post-hoc comparison), while no significant difference was observed for the remaining outcomes.

In order to adjust the observed results for possible confounders and assess the independent effect of brain sparing on FI and NEC development, we built specific multivariable GLM models, shown in Table 6. As in the previous GLM model, due to the significant collinearity observed between UA/DV-AREDF and the presence/absence of brain sparing (VIF > 6 for both covariates), only the brain sparing status was selected for inclusion in these GLMs; no collinearity issues were observed among the other variables included in the models (VIF < 2).

Compared to controls, FI prevalence was significantly increased in both AREDF infants with (OR = 3.153, 95% CI 1.682–5.907, *p* < 0.001) and without brain sparing (OR = 2.478, 95%CI 1.417–4.335, *p* = 0.001). Decreasing GA (OR = 1.268, 95% CI 1.155–1.392, *p* < 0.001) and formula feeding (OR = 1.884, 95% CI 1.172–3.029, *p* = 0.009) posed an additional, independent risk for this condition, while no effect was observed for the year of birth and antenatal steroid administration.

The risk of NEC (at any stage) was significantly increased in the presence of antenatal brain sparing (OR 2.845, 95% CI 1.188–6.813, *p* = 0.019), in formula-fed infants (OR 2.546, 95% CI 1.039–6.239; *p* = 0.041), and among those who required invasive respiratory support (OR 2.322, 95% CI 1.063–5.073, *p* = 0.035), while the remaining covariates showed no effect.

## 4. Discussion

The present study aimed to investigate the specific GI impact of different Doppler abnormalities in VLBW preterm infants, showing a significantly increased risk of developing adverse GI outcomes in relation to specific Doppler features.

According to our results, after the adjustment for several covariates, early Doppler alterations, such as UA-AREDF, are associated with a higher risk of developing FI and a subsequently longer time to reach FEF but not other GI complications, whereas more severe stages of Doppler impairment, such as UA+DV-AREDF, are associated with an increased risk of such ischemic complications as NEC and SIP. Moreover, brain-sparing remodeling was also demonstrated to be an independent risk factor for the development of both FI and NEC.

In the present study, infants with UA-AREDF and UA+DV-AREDF showed a 2- and 5-fold increase in the risk of developing FI, respectively, whereas antenatal evidence of brain-sparing remodeling was associated with a 3-fold increase in this risk compared to normal antenatal Doppler. The chronic fetal hypoxia observed in UA-AREDF and, even more, in UA+DV-AREDF fetuses and the brain sparing remodeling may alter the capacity of the splanchnic circulation to increase gut perfusion and oxygen delivery in response to the increased metabolic demand associated with enteral feeding, thus hindering the establishment of an adequate feeding tolerance. This is supported by postnatal evidence of reduced blood flow in the superior mesenteric artery, decreased gut oxygenation, and increased intestinal oxygen extraction in response to feed administration in preterm neonates with AREDF who developed FI [16,18]. A remarkable prevalence of FI in AREDF infants has also been reported by Aradhya et al., although they did not perform any comparisons between infants with or without Doppler abnormalities or with different Doppler features [25]. Ahamed et al. compared FEF time among SGA infants with and without evidence of abnormal antenatal Doppler [26]; after adjustment for GA and BW, the impact of Doppler abnormalities on FEF achievement was no longer significant. However, it is possible that the inclusion of several covariates that may be strictly correlated to antenatal Doppler impairment (e.g., BW and head circumference and length at birth) may have underpowered the multivariable analysis.

Consistently, time to achieve FEF and PN duration were significantly increased in the presence of UA-AREDF and, even more, of UA+DV-AREDF; to the best of our knowledge, the specific impact of UA+DV-AREDF on these variables has not been evaluated in other studies. It is possible that the longer time to achieve FEF observed in these groups reflected the higher prevalence of FI previously discussed. When the presence of brain sparing remodeling was selectively evaluated, however, we failed to demonstrate a significant impact of this condition either on the time needed to achieve FEF or on the duration of PN, despite the increased prevalence of FI observed in the brain sparing group. Conversely, Bozzetti et al. reported a longer time not only to achieve FEF but also to initiate minimal enteral feeds in UA-AREDF infants with evidence of brain sparing compared to those without brain-sparing evidence [17]. It is possible that the delayed introduction of enteral feeds in their population, which occurred on average at 5 days of life in relation to the gradual recovery of intestinal perfusion, may have influenced this data, whereas our local feeding protocol supports an early initiation of enteral feeds even in the AREDF population, using exclusively maternal or donor milk and strictly monitoring feeding tolerance. Nevertheless, it should be noted that the indication for human milk use and feeding tolerance monitoring are valid for all preterm infants admitted to our hospital, and that in our setting during the study period, the rates of feeding advancement were not differentiated “a priori” in AREDF infants but were rather modulated based on the individual feeding tolerance.

NEC is the most devastating intestinal complication occurring among preterm neonates. Over the last decades, growing knowledge of the underlying pathophysiological mechanisms has allowed for the development of a number of preventive strategies (e.g., human milk feeding, non-aggressive enteral protocols); nonetheless, the morbidity and mortality rates, along with the financial healthcare costs associated with this condition, are still remarkably high [15]. In our population, the prevalence of NEC (at any stage) at the univariate analysis was found to be significantly increased in the presence of antenatal UA+DV-AREDF and UA-AREDF. However, after adjustment for several covariates, the effect of UA-AREDF on NEC development (any stage) was not confirmed as significant. Conversely, the predisposing effect of brain-sparing remodeling on NEC development (at any stage) was confirmed significant by the multivariable analysis.

An increased risk of NEC in UA-AREDF infants has been previously reported in a meta-analysis of 14 observational studies, which described a 2-fold increase of NEC risk in AREDF infants compared to controls with normal Doppler [19], and in several later studies [4,18,20]. However, additional parameters, such as an altered DV flowmetry and brain sparing evidence, or other variables known to influence GI outcomes, such as the type of feeding, were not considered independent contributors to the increased NEC risk, probably due to the relatively small study samples. Moreover, the inclusion criteria and the adopted NEC definition varied across these studies.

Baschat et al. evaluated the prevalence of several neonatal outcomes, including NEC, among infants with UA-AREDF, UA-AREDF and brain sparing, and altered venous Doppler, which included both DV-AREDF and altered umbilical vein flowmetry and reflected an advanced deterioration of fetal haemodynamics with altered cardiac function, observing increased NEC rates in this latter group [2]. Conversely, Monogura et al. compared Doppler indices in AREDF infants who developed NEC vs. those who did not. When resistance indices were considered, an association between NEC development and increased vascular resistances in the UA, but not in the DV, was observed; on the contrary, when Doppler features were assessed as categorical variables, only increased pulsations in the umbilical vein, which are often observed in the final stages of fetal hemodynamic compromise, were found to be significantly associated with NEC development [13,21]. However, differently from the present data, where AREDF infants were compared to a control cohort with normal antenatal Doppler, Monogura et al. exclusively included infants with antenatal Doppler abnormalities, and this may have contributed to these different results.

In the present study, antenatal Doppler impairment with evidence of brain sparing was associated with a 2.5-fold increase in NEC risk compared to normal antenatal Doppler. Increased rates of composite adverse neonatal outcomes, including NEC, in IUGR infants with antenatal signs of vascular redistribution were also reported by the PORTO trials [27,28]; however, in these studies, NEC was not evaluated individually. Conversely, Baschat et al. found no significant difference in NEC rates between AREDF infants with and without evidence of brain sparing [2].

SIP shows different clinical and diagnostic features from NEC, as it generally occurs earlier and is characterized by focal necrotic areas, more often localized in the terminal ileum, while the remaining bowel appears grossly normal [24]. In our cohort, the prevalence of SIP was significantly increased in the cohort with impaired DV Doppler, while no cases were observed in the UA-AREDF group, and no significant differences were observed in relation to the brain sparing response. This is consistent with the greater hypoxia and acidemia that fetuses with DV-AREDF experience during this advanced phase of haemodynamic impairment, which may lead to the development of hypoxic-ischemic injury to the bowel mucosa even before birth [19]. Moreover, during early postnatal life, the metabolic responses to enteral feeding introduction enhance the oxygen demand at the gut level; however, due to the severe haemodynamic disturbances that are particularly pronounced in the presence of DV-AREDF may worsen the imbalance between intestinal oxygen delivery and consumption, thus further contributing to the development of focal bowel ischemia and subsequent SIP. An increased prevalence of SIP has been previously reported in preterm infants born by pre-eclamptic mothers; however, although pre-eclampsia is often associated with altered placental vascularization, the patterns of fetal Doppler were not specifically investigated [29]. Potential risk factors associated with SIP have been examined in several studies [30,31,32], however, none have assessed the possible role of antenatal Doppler abnormalities.

In line with previous evidence [2,33,34], we have observed significantly increased mortality rates in the UA+DV-AREDF group and in the presence of antenatal brain sparing, in line with the noxious consequences of the ensuing fetal haemodynamic compromise, which can persist during postnatal life.

According to the results of the multivariable GLMs, a lower GA and formula feeding are independent risk factors for adverse GI outcomes. The anatomical and functional immaturity of the bowel tract affect gut motility, alter the barrier function of the intestinal mucosa, and hinder the adequacy of digestive and absorptive processes [35], thus increasing the susceptibility of preterm infants to intestinal injury. Consistently with the present results, Kempley et al. investigated feeding tolerance and NEC prevalence in AREDF infants < and ≥29 weeks’ gestation, observing a significant increase in adverse GI outcomes in the more preterm subgroup [36]. According to their findings, different timings of enteral feeding introduction failed to effectively modify GI outcomes, whereas the use of exclusive breast milk during enteral feeding advancement significantly reduced the risk of FI and NEC. This is in line with the multiple protective effects of human milk and with the well-established negative role of formula feeding in NEC development, as extensively documented by longstanding evidence [37,38,39,40,41] and further confirmed by the present results. Hence, our findings highlight how the combination of increasing prematurity and placental vascular dysfunction with the following compromise of fetal hemodynamics significantly amplify the risk of adverse GI outcomes. Further data from prospective trials may aid in understanding whether tailored feeding strategies, such as the mandatory use of exclusive human milk for enteral feeding introduction and advancement in preterm infants with antenatal Doppler impairment, are effective.

In our cohort, infants with antenatal Doppler abnormalities received a complete course of antenatal steroids more frequently than controls, consistent with the closer surveillance of these pregnancies [42]. The effect of antenatal steroids on NEC is controversial. While Roberts et al. in their meta-analysis reported a reduced neonatal mortality and morbidity, including NEC incidence [43], no significant difference in the prevalence of NEC and of other morbidities was more recently reported in the meta-analysis by Blankenship et al. comparing SGA preterm infants between SGA preterm infants who received steroid prophylaxis to those who did not [44]. In order to consider this potential confounder, we included antenatal steroids administration as a covariate in the multivariable GLM analysis, confirming the independent increase of FI and NEC risk in the presence of specific antenatal Doppler abnormalities, despite the increased prevalence of complete steroid prophylaxis associated with these conditions, while no effect of this prophylaxis on FI and NEC was observed, consistently with Blankenship et al. [44].

A strict monitoring of Doppler flowmetry may help to determine the optimal timing of delivery in high-risk pregnancies [45,46]. The detection of altered flowmetry in the UA does not represent a current indication for iatrogenic delivery but poses a warning for careful monitoring of the pregnancy [9]. Instead, the evidence of Doppler alterations in the DV poses a significant challenge to the fetal health, as each day in utero doubles the odds of stillbirth independently of GA [33]. Hence, current guidelines recommend an urgent iatrogenic delivery in the presence of an abnormal DV Doppler [42]. This may explain the lower GA observed in the UA+DV-AREDF group compared with the UA-AREDF group; however, the results of the multivariable analysis have confirmed the independent role of an altered DV flowmetry in increasing GI risks, regardless of GA.

In the present cohort, the number of infants with major GI complications such as NEC stage ≥ 2a and SIP was small and thus represents a potential study limitation. In order to determine the number of these infants, we analyzed data from a 12-year period. The retrospective nature of this study and the advances that occurred in obstetrics and neonatal practice during this study period may have influenced the observed results; to address this potential bias, the year of birth was included in the GLMs, ruling out a possible effect of this variable on the study outcomes. Moreover, the Apgar score at 5 minutes and the need for invasive ventilation were also included in the multivariable analysis, as they may act as potential indicators of clinical severity.

The relatively small number of infants with AREDF, especially with altered DV velocimetry, also needs to be acknowledged among the study limitations, as it did not allow to include both UA/DV Doppler status and brain sparing in a unique and adequately powered multivariable analysis. Moreover, despite the efforts made to adjust the observed results for possible confounding or influencing factors, hidden confounders or biases are still possible. A targeted multicenter trial, based on a larger cohort, may allow to validate the present results and to merge the two grouping systems into a unique classification based on four groups (UA-AREDF, brain sparing; UA-AREDF, no brain sparing; UA+DV-AREDF, brain sparing; UA+DV-AREDF, no brain sparing).

To the best of our knowledge, this is the first study investigating the impact of specific antenatal Doppler abnormalities on multiple GI outcomes. Indeed, most of the available literature on this topic either uses SGA as a proxy for antenatal AREDF or assesses composite outcomes mixing GI conditions such as NEC with neonatal complications related to different organs or systems. Although the term SGA is often used as a synonym for IUGR, it includes both constitutively small infants and those who have not reached their genetically determined growth potential due to an impairment of placental vascularization. The present results, based on an accurate evaluation of antenatal Doppler data, demonstrate how a considerable proportion of AREDF infants had a BW > 10° percentile, thus being excluded by the SGA definition, while nearly 1 out of 6 infants with normal antenatal Doppler were SGA. Hence, the use of the SGA definition in place of an accurate assessment of antenatal Doppler features may under-represent the AREDF population, thus possibly affecting the accuracy of the performed assessments and contributing to the heterogenicity of the currently available literature.

Placental vascular dysfunction significantly influences postnatal GI outcomes in preterm infants. A growing severity of the fetal haemodynamic compromise associated with this condition leads to a greater risk of adverse GI sequelae, adding to the noxious effects of intestinal immaturity associated with preterm birth. Hence, a careful assessment of antenatal Doppler features is of particular importance to estimate the individual intestinal risk of preterm neonates and to identify those at highest risk who would benefit from tailored management of enteral feeding, including the use of exclusive breast milk and strict monitoring of their feeding tolerance, to reduce the clinical and healthcare burden of GI complications.

## Figures and Tables

**Table 1:** Clinical characteristics in infants with UA-AREDF, UA+DV-AREDF and controls. Significant between-group comparisons are highlighted in bold. Abbreviations: BW: birth weight; GA: gestational age; IQR: interquartile range; SGA: small for gestational age; UA-AREDF: absent or reversed end diastolic flow in the umbilical artery only; UA+DV-AREDF: absent or reversed end diastolic flow in the umbilical artery and in the ductus venosus.

Clinical Characteristics	UA-AREDF (*n* = 97)	UA+DV-AREDF (*n* = 27)	Controls (*n* = 446)	*p*-Value
GA (weeks), median (IQR)	31 (29–32) *^§^	29.1 (27.7–30.3) *	30.1 (27.7–31.6) ^§^	**0.001**
BW (g), median (IQR)	1069 (850–1274) °^§^	825 (547–1033) °*	1309 (994–1480) *^§^	**<0.001**
BW Z-score, median (IQR)	−1.35 (−1.80; −0.91) *	−1.37 (−1.95; −1.10) ^§^	0.17 (−0.73; 0.69) *^§^	**<0.001**
SGA, n (%)	52 (53.6)	14 (51.9)	58 (13.0)	**<0.001**
Length (cm), median (IQR)	37 (34–39) *	33.5 (31–36) ^§^	39 (35–40) *^§^	**<0.001**
Head circumference (cm), median (IQR)	27 (25–28)	25 (23–27.5) *	28 (26–29) *	**<0.001**
Sex (males), n (%)	50 (51.5)	13 (48.1)	231 (51.8)	0.934
Twins, n (%)	28 (28.9)	11(40.7)	159 (35.7)	0.356
Antenatal steroids (complete course), n (%)	84 (86.5)	17 (63)	320 (71.7)	**0.002**
Maternal hypertension, n (%)	46 (47.4)	9 (33.3)	92 (20.6)	**<0.001**
C-section, n (%)	97 (100)	27 (100)	374 (83.9)	**<0.001**
Any formula feeding during hospital stay, n (%)	76 (78.4)	14 (51.9)	344 (77.1)	0.096

*°^§^ significant pairwise comparisons at Bonferroni post-hoc test. Post-hoc *p*-values are specified in the main text.

**Table 2:** Gastrointestinal and nutritional outcomes in infants with UA-AREDF, UA+DV-AREDF, and controls. Significant between-group comparisons are highlighted in bold. Abbreviations: FEF: full enteral feeding; FI: feeding intolerance; IQR: interquartile range; NEC: necrotizing enterocolitis; PN: parenteral nutrition; SIP: spontaneous intestinal perforation; UA-AREDF: absent or reversed end diastolic flow in the umbilical artery only; UA+DV-AREDF: absent or reversed end diastolic flow in the umbilical artery and in the ductus venosus.

Outcome	UA-AREDF (*n* = 97)	UA+DV-AREDF (*n* = 27)	Controls (*n* = 446)	*p*-Value
FI, n(%)	40 (41.2)	18 (66.7)	126 (28.3)	**<0.001**
NEC (any stage), n (%)	11 (11.3)	7 (25.9)	35 (7.8)	**0.005**
NEC (≥2a Bell’s stage), n (%)	5 (5.2)	2 (7.4)	10 (2.2)	0.119
SIP, n (%)	0 (0.0)	4 (14.8)	6 (1.3)	**<0.001**
Mortality, n (%)	1 (1)	4 (14.8)	1 (0.2)	**<0.001**
Time to FEF (days), median (IQR)	22 (16–31)	24 (20–47) *	20 (14–31) *	**0.038**
PN duration (days), median (IQR)	19 (13–27)	23 (20–42) *	17 (11–30) *	**0.011**
Length of hospitalization (days), median (IQR)	44 (34–69) °	61 (53–104) °*	42 (29–69) *	**0.003**

*° significant pairwise comparisons at Bonferroni post-hoc test. Post-hoc *p*-values are specified in the main text.

**Table 3:** Results of the generalized linear models for outcomes that were significantly associated with the severity of antenatal Doppler alterations in the univariate analysis. B indicates the regression coefficients for continuous data outcomes that can be interpreted as in a linear regression model, whereas OR indicates the odds ratio for binary outcomes. When the independent variable is categorical, one group is used as the reference category (§). Significant *p*-values are highlighted in bold. Abbreviations: CI, confidence interval; FEF: full enteral feeding; UA-AREDF: absent or reversed end diastolic flow in the umbilical artery only; UA+DV-AREDF: absent or reversed end diastolic flow in the umbilical artery and in the ductus venosus.

	Outcome	Time to FEF				Feeding Intolerance				Necrotizing Enterocolitis			
		B	95% CI		*p*-Value	OR	95% CI		*p*-Value	OR	95% CI		*p*-Value
Variable			Lower	Upper			Lower	Upper			Lower	Upper	
UA+DV-AREDF		15.529	8.991	22.066	**<0.** **001**	5.622	2.230	14.170	**<0.** **001**	4.311	1.425	13.044	**0.** **010**
UA-AREDF		4.385	0.848	7.922	**0.** **015**	2.281	1.387	3.749	**0.001**	1.599	0.729	3.507	0.241
Normal antenatal Doppler ^§^		0				1				1			
Antenatal steroids, complete course		0.037	−3.033	3.107	0.981	0.816	0.522	1.277	0.374	1.580	0.716	3.485	0.257
Antenatal steroids, incomplete course or not given ^§^		0				1				1			
Any formula feeding during hospital stay		2.462	−0.617	5.541	0.117	1.953	1.207	3.161	**0.** **006**	2.725	1.090	6.807	**0.** **032**
Exclusive human milk feeding during hospital stay ^§^		0				1				1			
Invasive respiratory support		11.286	7.635	14.936	**<0.001**	0.966	0.570	1.638	0.897	2.409	1.094	5.306	**0.029**
Non-invasive respiratory support ^§^		0				1				1			
Gestational age (decreasing weeks		2.894	2.279	3.509	**<0.001**	1.262	1.149	1.385	**<0.001**	1.099	0.953	1.267	0.195
Apgar score at 5 minutes		0.416	−0.772	1.605	0.492	1.140	0.958	1.357	0.140	1.086	0.843	1.399	0.524
Year of birth		0.001	−0.370	0.371	0.998	0.966	0.915	1.020	0.208	0.947	0.870	1.030	0.203

**Table 4:** Clinical characteristics in infants with abnormal antenatal Doppler with and without antenatal evidence of brain sparing and controls. Significant between-group comparisons are highlighted in bold. Abbreviations: BW: birth weight; GA: gestational age; IQR: interquartile range; SGA: small for gestational age.

Clinical Characteristics	Abnormal Doppler, No Brain Sparing (n = 70)	Abnormal Doppler, Brain Sparing (n = 54)	Controls (n = 446)	*p*-Value
GA (weeks), median (IQR)	30.9 (29–32.1) *	30 (28.7–31.6)	30.1 (27.7–31.6) *	**0.033**
BW (g), median (IQR)	1088 (840–1265) *	922 (731–1205) ^§^	1309 (994–1480) *^§^	**<0.001**
BW Z-score, median (IQR)	−1.40 (−1.75; −0.87) *	−1.29 (−1.97; −1.05) ^§^	0.17 (−0.63; 0.79) *^§^	**<0.001**
SGA, n (%)	39 (55.7)	27 (50)	58 (13)	**<0.001**
Length, median (IQR)	37 (34–39) *	36 (33–39) ^§^	39 (35.5–40) *^§^	**<0.001**
Head circumference, median (IQR)	27 (25–28)	26 (24–28) *	28 (26–29) *	**0.001**
Sex (males), n (%)	34 (48.6)	29 (53.7)	231 (51.8)	0.836
Twins, n (%)	19 (27.1)	20 (37)	159 (35.7)	0.355
Antenatal steroids (complete course), n (%)	58 (82.9)	43 (79.6)	320 (71.7)	**0.010**
Maternal hypertension, n (%)	31 (44.3)	24 (44.4)	92 (20.6)	**<0.001**
C-section, n (%)	70 (100)	54 (100)	374 (83.9)	**<0.001**
Any formula feeding during hospital stay, n (%)	56 (80)	34 (63)	344 (77.1)	**0.190**

*^§^ significant pairwise comparisons at Bonferroni post-hoc test. Post-hoc *p*-values are specified in the main text.

**Table 5:** Gastrointestinal and nutritional outcomes in infants with abnormal antenatal Doppler with and without brain sparing and controls. Significant between-group comparisons are highlighted in bold. Abbreviations: FI: feeding intolerance; FEF: full enteral feeding; IQR: interquartile range; NEC: necrotizing enterocolitis; PN: parenteral nutrition; SIP: spontaneous intestinal perforation.

Outcomes	Abnormal Doppler, No Brain Sparing (n = 70)	Abnormal Doppler, Brain Sparing (n = 54)	Controls (n = 446)	*p*-Value
FI, n (%)	31 (44.3)	27 (50)	126 (28.3)	**<0.001**
NEC (any stage), n (%)	8 (11.4)	10 (18.5)	35 (7.8)	**0.031**
NEC (≥2a Bell’s stage), n (%)	4 (5.7)	3 (5.6)	10 (2.2)	0.143
SIP, n (%)	1 (1.4)	3 (5.6)	6 (1.3)	0.082
Mortality, n (%)	2 (2.8)	3 (5.6)	1 (0.2)	**<0.001**
Time to FEF (days), median (IQR)	19 (16–33)	24 (18–33)	20 (14–31)	0.127
PN duration (days), median (IQR)	18 (13–31)	22 (16–29)	17 (11–30)	0.086
Length of hospitalization (days), median (IQR)	42 (34–73)	58 (43–73)*	42 (29–69) *	**0.020**

* significant pairwise comparisons at Bonferroni post-hoc test. Post-hoc *p*-values are specified in the main text.

**Table 6:** Results of the generalized linear models for outcomes that were significantly associated with the presence of brain sparing in the univariate analysis. When the independent variable is categorical, one group is used as the reference category (§). Significant *p*-values are highlighted in bold. Abbreviations: CI, confidence interval; OR, odds ratio.

	Outcome	Feeding Intolerance				Necrotizing Enterocolitis			
		OR	95% CI		*p*-Value	OR	95% CI		*p*-Value
Variable			Lower	Upper			Lower	Upper	
Abnormal Doppler, brain sparing		3.153	1.682	5.907	**<0.001**	2.845	1.188	6.813	**0.** **019**
Abnormal Doppler, no brain sparing		2.478	1.417	4.335	**0.001**	1.561	0.638	3.818	0.329
Normal antenatal Doppler ^§^		1				1			
Antenatal steroids, complete course		0.789	0.507	1.229	0.295	1.465	0.672	3.195	0.337
Antenatal steroids, incomplete course or not given ^§^		1				1			
Any formula feeding during hospital stay		1.884	1.172	3.029	**0.** **009**	2.546	1.039	6.239	**0.** **041**
Exclusive human milk feeding during hospital stay ^§^		1				1			
Invasive respiratory support		0.956	0.565	1.616	0.866	2.322	1.063	5.073	**0.035**
Non-invasive respiratory support ^§^		1				1			
Gestational age (decreasing weeks)		1.268	1.155	1.392	**<0.001**	1.109	0.963	1.278	0.149
Apgar score at 5 minutes		1.131	0.952	1.344	0.162	1.078	0.839	1.384	0.559
Year of birth		0.963	0.913	1.016	0.169	0.941	0.864	1.025	0.161

## Data Availability

The study data are available upon reasonable request from the corresponding author.
